# A Shared Mealtime Approach to Improving Social and Nutritional Functioning Among Older Adults Living Alone: Study Protocol for a Randomized Controlled Trial

**DOI:** 10.2196/resprot.4050

**Published:** 2015-04-21

**Authors:** Joanna McHugh, Olga Lee, Niamh Aspell, Brian A Lawlor, Sabina Brennan

**Affiliations:** ^1^NEIL ProgramInstitute of NeuroscienceTrinity College DublinDublinIreland

**Keywords:** nutrition, randomized controlled trial, older, aging, intervention, social support, anthropometry, social cognitive theory

## Abstract

**Background:**

Older adults living alone are at increased risk of malnutrition as well as social isolation. Previous research has evaluated psychosocial interventions aimed at improving social support for older adults living alone. One meta-analysis in particular has suggested that multimodal psychosocial interventions are more effective than unimodal interventions. As such, it may be more effective to deliver an intervention which combines nutritional and social support together. Consequently, we designed the RelAte intervention, which focuses on shared mealtimes as a source of combined social and nutritional support for older adults living alone who are at risk of social isolation.

**Objective:**

The objective of the RelAte trial was to evaluate the impact of such an intervention on energy intake, anthropometric measurements, and nutritional social cognitive variables among older adults living alone in the community.

**Methods:**

There are 100 participants that will be recruited and randomized to either the treatment (n=50) or the control group. The treatment group will receive a visit from a trained peer volunteer once weekly for a period of 8 weeks. Outcomes of interest include: energy intake, social cognitive factors related to diet, abdominal circumference, body mass index, psychosocial well-being, frailty, nutritional status, and health utilities. Outcomes will be obtained at baseline, immediately postintervention (8 weeks after baseline), 12-week follow-up, and 26-week follow-up by assessors blinded to participants’ randomized assignment.

**Results:**

The Relate trial is currently active. We are currently at data analysis stage. The study started in June 2013 and will run until June 2015.

**Conclusions:**

Results from this study will primarily describe the effectiveness of a shared mealtime intervention for older adults living alone in terms of their dietary well-being, physical health, and psychosocial well-being.

**Trial Registration:**

Trial Registration: Clinicaltrials.gov NCT02007551; http://clinicaltrials.gov/ct2/show/NCT00102401 (Archived by WebCite at http://www.webcitation/6WptuVTtz).

## Introduction

### Socially Isolated Older Adults

Older adults who live alone independently in the community are at increased risk of becoming socially isolated, relative to those who live with others [1,2]. Social isolation, in turn, is a significant risk factor for the development of cognitive impairment, as well as increased risk of dementia, morbidity of other types, and mortality [3-5]. Since social isolation has such a significant effect on older adults, interventions are recommended. To date, there have been many attempts to design and evaluate effective and scalable interventions to improve social support among older adults. The most common form of social support intervention is the “befriending” intervention [6], whereby volunteers are trained to provide emotional support and reciprocal social support for the participant. Befriending interventions can be effective in improving psychosocial functioning and reducing depressive symptomatology [7].

### Mealtime Interventions

Social isolation among older adults living alone can also increase the risk of malnutrition [8-11]. The state of malnutrition itself is associated with increased morbidity, mortality, and up to 20 times more complications at hospitalization. While studies have in the past focused on mealtime interventions, these interventions have tended to focus on institutionalized populations, or older adults with dementia [12-20]. The concept of a mealtime intervention does not appear to have been applied to a healthy independently living older population. However, the concept of a mealtime is gaining ground as a scientifically interesting topic of research [21], particularly the idea of shared mealtimes as opposed to eating alone [22]. A study of 150 older adults found that 32% never shared a meal with others [23].

It is important that interventions are designed according to a theoretical framework, to inform the research design, the content of the intervention, and to facilitate interpretation of results [24,25]. A popular theoretical framework underlying many psychosocial interventions is Social Cognitive Theory [26,27]. Interventions designed in accordance with this theory focus on the individual as learning most effectively from others, particularly when learning health behaviors. Social cognitive interventions aim to facilitate the transmission of knowledge from one individual to another, ideally between peers, so as to improve self-efficacy and ultimately change behavior. Outcome expectations, or the belief that a particular outcome will follow from a behavior, are also of interest as an outcome variable in social cognitive interventions.

A meta-analysis of befriending interventions performed by Cattan et al [28] concluded that active interventions, whereby some effort on the part of the participant was required, were more effective than those without. Furthermore, since challenges are rarely faced in isolation, Sabir et al have recommended that successful interventions should target more than one challenge at a time [29]. Combining social and nutritional support may have synergistic effects on nutritional outcomes since companionship may improve energy intake at meals [10,30]. Furthermore, social support appears to be best received if combined with a secondary leisure activity [31], presumably because incidental social contact is more acceptable to older adults than purposeful contact, which may highlight a stigmatized condition of social isolation [32]. Thus, we decided to target nutritional and social challenges faced by older adults living alone in combination, using the RelAte shared mealtime intervention. The purpose of this trial is therefore to examine the preliminary effectiveness of implementing the RelAte shared mealtime intervention for older adults living alone at risk of social isolation.

## Methods

### Study Design

The RelAte trial is a parallel assignment, assessor-blinded, randomized controlled trial aimed at evaluating the effectiveness of an 8-week peer-delivered social and nutritional intervention relative to text-based educational information for older adults who are living alone and at risk of social isolation. The trial protocol was registered on an Internet trial database [33]. The primary aim of the trial is to indicate the effectiveness of the RelAte intervention and its impact on social cognitive and anthropometric factors for older adults living alone independently in the community. A total of 100 participants will be recruited to the study, age 60 and over, and will be randomly assigned to the treatment or the control group based on a minimization procedure [34]. The study is a feasibility trial, and as such, our target sample size is not aimed at achieving sufficient power to make conclusive statements about the intervention effectiveness, but rather to obtain sufficient effect sizes to allow preliminary statements to be made about the impact of such an intervention, and to inform further, optimally powered, trial studies evaluating the intervention.

### Participants

Participants for the current study are eligible if they are 60 years of age or older, living alone, and deem themselves to be at risk of social isolation. A phone screen will then determine whether participants are eligible for inclusion in the study according to the following criteria: that they show no sign of cognitive impairment as defined using the Telephone Cognitive Screen [35]; that they do not report a history of stroke, epilepsy, schizophrenia, bipolar affective disorder, recurrent psychotic depression, or alcohol or drug abuse within the past 5 years; that they do not report the use of anticonvulsants or antipsychotic medications; that they do not have significant hearing difficulties which are not resolved using a hearing aid; that they do not report a history of any illness which caused permanent decrease in memory or other cognitive functions; and that they do not currently have any bloodborne, airborne, or contact-borne infectious diseases which would threaten the well-being of the peer volunteer. In the original protocol plan, an additional exclusion criterion was that eligible participants must have scored as socially isolated on the Lubben Social Network Scale [36], but following the screening of the first 10 participants, it was found that this yielded only 20% eligibility from interested participants; since older adults living alone are a particularly difficult group to recruit for research without this additional criterion, it was deemed necessary to instead change this criterion to include participants if they themselves felt that they were at risk of becoming socially isolated.

### Recruitment

Recruitment strategies include: mail-drops to sheltered accommodation areas in the greater Dublin area; presentations to senior citizens groups, active retirement groups, and other social groups for older adults; publications in national newspapers and use of national television media; published recruitment advertisements in parish newsletters; recruitment via day centers and clinics; recruitment via public health nurse networks and other allied health professionals working in the community; disseminating fliers to primary care offices and pharmacies; and word of mouth.

### Informed Consent

Approval of the trial protocol and consent forms has been obtained by the School of Psychology Research Ethics Committee, Trinity College Dublin (Project, RelAte 12122-2013) prior to the recruitment of any participants. All participants must provide informed written consent prior to their involvement with the study.

### Measures

All measures will be obtained at baseline, 8-weeks postbaseline (ie, immediately after the 8-week intervention period), 12-week follow-up, and 26-week follow-up, for all 100 participants. Assessments will be conducted by research assistants with qualifications in psychology, who will be blinded to the randomized assignment of the participants.

### Primary Outcome Measures

#### Social Cognitive Variables

These variables describe the self-efficacy, self-regulation, outcome expectations, and social support related to dietary behavior for participants. The Generalised Self-Efficacy Scale [37], Nutrition Self-Efficacy Scale [38], and Health Beliefs Survey [39] will all be used to evaluate these variables across all participants, across the four measurement points.

#### Energy Intake

Energy intake in kilocalories will be assessed using two 24-hour dietary recalls, whereby participants are asked to recall in detail everything they ate and drank over the previous 24-hour period. The assessors will receive dietetic training in administering these dietary recalls. The dietary recalls will be converted to kilocalorie values using Nutritics software [40].

### Secondary Outcome Measures

#### Quality of Life

Participant quality of life will be measured at each timepoint using the 19-item Control, Autonomy, Self-Realisation & Pleasure Scale [41].

#### Cognition

Participant cognition over time will be measured using the Montreal Cognitive Assessment [42] and the Trail Making Test [43]; premorbid cognitive function will be assessed using the National Adult Reading test at baseline only [44].

#### Social Connectedness

The Berkman-Syme Social Network Index will be used to measure social connectedness over time [45].

#### Loneliness

The De Jong Gierveld scale will be used to assess loneliness over time [46].

#### Psychological Well-Being

Psychological well-being will be assessed across participants over time using the Centre for Epidemiological Studies depression scale [47], the Hospital Anxiety & Depression Anxiety subscale [48], and the Ryff scale of well-being [49].

#### Nutrition

Nutritional health will be assessed using the Mini Nutritional Assessment [50] and the Food Enjoyment Scale [51].

#### Physical Health

Abdominal circumference will be measured using a measuring tape; body mass index will be measured using weight (kg) and height (cm) readings from a clinical stadiometer and body composition weighing scales. Grip strength and overall frailty will be assessed and operationalized according to the Survey of Health, Ageing, and Retirement in Europe frailty instrument [52], and overall health will be assessed using the Health Utilities Index [53].

### The Intervention

The RelAte intervention is an 8-week, multicomponent peer-delivered intervention, which is delivered in the home of the participant. The intervention each week consists of a visit from a matched peer (matched based on location and gender), who decides with the participant on a recipe from the RelAte guidebook. These recipes were chosen based on the ease of preparation for one person (since the RelAte participants live alone) and for cost-effectiveness also. Each week, the participant and volunteer choose a recipe, and the volunteer brings the ingredients to the home of the participant, so that they can together prepare and share a meal. The volunteers have all received basic culinary and nutritional training and, in keeping with recommendations for active interventions, participants are advised that they must engage with all steps of the cooking process, insofar as they are able. Since the intervention is based on Social Cognitive Theory, there must be opportunities for vicarious learning (through watching the volunteer cook), social support provided by the volunteer, opportunities for skill mastery (by helping with the cooking), and facilitation of goal setting if the participant wishes. The weekly visits last for 90 minutes and, aside from the guidelines listed here, are unstructured. The participants in the control group receive the RelAte guidebook, which contains recipes and nutritional/culinary information and advice, but no visitor.

### Planned Analyses

We hypothesize that engagement with the RelAte intervention will result in increased energy intake for individuals with less than ideal intake, and decreased energy intake for those with higher than ideal intake. We hypothesize that self-regulation, self-efficacy, outcome expectations, and social support relating to dietary behavior will all improve over time for the treatment group relative to the control group. A weighted analysis of covariance model with appropriate covariates of baseline measures and age, baseline social isolation, and gender will be used unless there are significant issues with missing data and unbalanced observations, in which case mixed models analyses will be used. All analyses will follow the intention-to-treat principle. In dealing with missing data, multiple imputation methods will be used.

## Results

The Relate trial is currently active. We are currently at data analysis stage. The study started in June 2013 and will run until June 2015.

## Discussion

To summarize, the RelAte trial is a two-arm, assessor-blind, parallel randomized controlled trial, which aims to evaluate the effectiveness of an 8-week combined social and nutritional mealtime intervention for older adults living alone who self-report to be at risk of social isolation. We hypothesize that engagement with the intervention, which is peer-led and modelled upon Social Cognitive Theory principles, will result in improved self-regulation, self-efficacy, outcome expectations, and social support pertaining to dietary behavior, as well as improving energy intake among older adults living alone. The study is an opportunity also to evaluate dietary behavior and intake among older adults who live alone, since this type of research has rarely been conducted with an independent Irish older population. It is expected that recruitment issues may hinder study progress, since older adults living alone are reportedly a difficult demographic to recruit for research. Furthermore, since older adults living alone are at increased morbidity risk, it is possible that the later measurement points of the study will be subject to a high attrition rate, although this is unavoidable with this population. Similarly, adherence to the weekly 8-week intervention may be poor, due to unavoidable health-related issues. Findings will not be conclusive since the sample size is not based on a power calculation; however, findings will indicate the preliminary effectiveness of a shared mealtime intervention, and hopefully elucidate future directions for research in this area.
